# Inequality in the Face of Death: The Income Gradient in Mortality of the Spanish Pre-Recession Working-Age Population

**DOI:** 10.3390/ijerph182312379

**Published:** 2021-11-25

**Authors:** Pedro García-Castrillo, María A. González-Álvarez

**Affiliations:** Department of Economic Analysis, University of Zaragoza, 50005 Zaragoza, Spain; pgarcia@unizar.es

**Keywords:** income, determinants of health, mortality rate

## Abstract

The purpose of this paper is to evaluate the association between socioeconomic status (SES) and mortality over a three-year period for working-age Spaniards (2007–2009), paying particular attention to the effect of income level. The analysis is relatively new in Spain, and the studies are limited. Neither income nor wealth are included in existing Spanish mortality studies. The main reason for this limitation is the nature of the data sets used, mainly Census Records. We overcome this problem by using data on 693,994 individuals taken from a Social Security sampling and used to estimate the probabilities of death for each income decile and the mortality rate ratios in three different models: (1) using only income, controlled by age and sex, (2) adding socio-economic and geographical variables, and (3) adding level of education. However, the data used here also have some limitations. They do not include government employees, the military or the Department of Justice personnel, whose exclusion we believe causes an under-representation of highly educated people in our sample. The results confirm that there is a non-linear relationship between mortality and income. This non-linear relationship implies that income redistribution resulting from progressive taxation systems could lead to higher reductions in mortality for low-income groups than the reductions induced in the mortality of the high-income population, thus reducing overall mortality.

## 1. Introduction

There seems to be a strong consensus on the existence of a relationship between socioeconomic status (SES) and mortality. However, the measurements or dimensions of SES vary, and the consensus is weaker when addressing the importance of each dimension and the causal mechanisms governing the interaction between SES and mortality. Cutler et al. [1] group SES indicators into four dimensions (education, financial resources, hierarchy and race/nationality) and explore the mechanisms or pathways by which each of them interact with mortality. It is necessary to shed some light on the empirical literature and, therefore, supranational and individual country studies have proliferated in recent years. However, in Spain such studies have been limited with regard to both the SES indicators used and the geographical area covered.

The main reason is the absence of data combining information on mortality and the various indicators that can be included under the generic term of socioeconomic status. In the case of Spain, the standard practice has been to jointly use Census records and mortality data from the National Institute of Statistics (INE). However, deficiencies in the mortality registers of some provinces have limited the spatial extent of the analysis using these sources of data. Furthermore, the absence of adequate socioeconomic information has conditioned the selection of representative instruments for the SES, which has been limited to two variables: the level of education and, in some cases, the occupational class. Neither income nor wealth are included in mortality studies in Spain.

With the aforementioned limitations, numerous reports have been written on the socioeconomic inequalities in mortality in Spain. Some papers have been included in European studies [2,3,4]. Two characteristics are common in the majority of these reports: they are limited to urban areas (Barcelona) and/or regions (Basque Country and Madrid), and they use the level of education as the only economic indicator. The results conclude that the effect of socioeconomic inequality on mortality in Spain is relatively low in comparison to the rest of Europe. Other papers suffer from limitations on the SES indicator used and refer to specific geographical locations. Except for one national study, the rest are restricted to Madrid [5,6], to Barcelona [7,8] or to both [9]. The use of occupation as a representative economic indicator is much more limited [10,11,12]. Therefore, the relationship between mortality and income in Spain remains an unexplored field. 

Income is clearly related to health, as is access to adequate climatized housing, the possibility of selecting neighborhoods with less pollution, acquiring healthy food, or purchasing medicine and health care. Good health also increases the possibility of entering the labor market. Conversely, ill health can lead to leaving a job, reducing working hours or doing part-time work, all of which affect income and professional careers both in the long and the short term. This implies the existence of an inverse causality between health and income. This is one of the reasons why there is a debate on which variable best measures the effect of socioeconomic inequalities in mortality. Without entering this debate, we believe that all the variables mentioned interact and include some facet of the effect of socioeconomic disparities on mortality. 

Reports are becoming more conclusive on the existence of the relationship between mortality and income, on the nature of the non-linear gradient, and on the attenuation (total or partial) of the relationship when it is controlled by other types of socioeconomic variables, including level of education. Such studies have been carried out in the U.S. [13,14,15], in Norway [16,17], in Finland [18,19], in New Zealand [20], and in European countries [3]. 

The nature of the relationship between mortality and income is relevant to the health outcomes of redistributive policies. The effects of this relationship on life expectancy have a direct effect on an aspect of our reality that is the subject of much social debate. This is the question of the sustainability of public pension systems, which has prompted reform processes in which one of the key variables is the increase in the retirement age, based on the increase in life expectancy. If inequalities in mortality have a social and economic character, this means that life expectancy is not the same for all. It varies according to social status. Thus, the generalized raising of the retirement age can lead to the paradox that low-income sectors have long periods of contributions (normally with little formal education and therefore they start working earlier) and, if their life expectancy at retirement age is short, their contributions end up financing those with a high economic status whose life expectancy is longer.

This paper explores these questions by studying the relationship between income and mortality in the working-age population of Spain before the Great Depression, during the years 2007 to 2009. This work focuses on income mainly because there are existing studies on the relationship between education and mortality in Spain, while studies on the relationship with income are lacking. This does not exclude the possibility of including education in the analysis to determine how it affects the relationship between income and inequality in the probability of death. 

For this analysis, data on 693,994 individuals were taken from a social security sample and used to estimate the probabilities of death for each income decile, and the mortality rate ratios for three different models: (1) using only income as an SES indicator, controlled by age and sex, (2) adding other socio-economic and geographical variables and, (3) adding level of education. This estimation strategy allows us to analyze whether, as some of the literature on the subject suggests, the inclusion of other socioeconomic indicators attenuates the relationship between income and the probability of death. The remainder of the paper is organized as follows. Section 2 describes the data, methodology and analysis framework. Section 3 presents the empirical results of the three estimated models. Finally, Section 4 draws the most important conclusions and identifies the economic implications.

## 2. Materials and Methods

### 2.1. Data

The data source is the Continuous Working Life Sample (CWLS), whose usefulness in the field of health economics has been pointed out by several studies, although its use has been essentially directed to the field of labor economics [21,22]. The sampling is based on those individuals who had dealings with the Social Security system in the year of reference (2007), whether they were working or receiving some type of benefit or pension. On this set of individuals, simple random sampling is carried out on 4% of the population (1,200,076 people in 2007). Over the three years analyzed, some individuals disappear from the sample and are replaced by others. One of the reasons for abandoning the sample is death, and the records indicate the time of decease. The CWLS has some limitations. It does not include unemployed people who did not receive benefits in the year of reference, nor those affiliated with other provider systems, such as government employees, the military, and the Department of Justice. This includes around a million people from specific state entities whose exclusion we believe causes an underrepresentation of highly educated people in our sample.

The data relate to those individuals whose ages range from 25 to 64 years old, who have some form of income and have no missing data in all the other variables. The number of individuals is 693,994 and the number of deaths over the years 2007–2009 is 3415, whose distribution by age and sex is presented in Table 1. A comparison with the Spanish Labor Force Survey reveals a slight over-representation in our sample of the older group of individuals with a corresponding under-representation of the rest. The reason is that our sample includes non-active individuals who receive early retirement or disability benefits, being more numerous in advanced ages.

### 2.2. Level of Education

Given the aforementioned limitations, there is an under-representation of people with higher education degrees in the CWLS, mainly due to the exclusion of significant collectives of the population of reference working in specific state entities, the majority of whom have university degrees. There are four levels of education. O is assigned to university degrees, 1 to post-secondary school studies, 2 to ESO (Obligatory Secondary Education, which is from age 12 to age 16), and 3 to uncompleted primary studies and those who are illiterate. Differences in educational level are very sensitive to age group. Among the younger population, there are hardly any illiterates, while among the elderly, the majority of the population has low levels of education (Table 2). It is interesting to note that the educational level of the youngest group (25–34 years) is lower than that of the previous generation (35–44). It is possible that the late incorporation into the labor market of the younger generation with university degrees is the cause of the under-representation of this group in the sample.

### 2.3. Income

All entities that pay salaries, pensions or unemployment benefits are obliged to present an annual summary of tax withholdings from all people who have any source of income that is subject to IRPF (payroll taxes) (excluding the Basque Country and Navarra, which have a special tax regimes). Income from savings is not included. 

**Figure 1:**
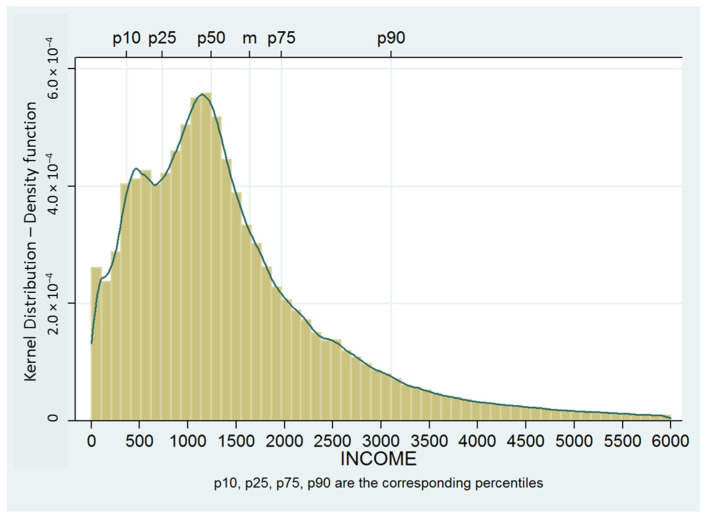
Monthly income distribution (kernel distribution). P10, p25, p50, p75 and p90 are the corresponding percentiles.

For each person, there are as many records as there are different tax withholders they have had throughout the year. The different sources of income have been classified into four main groups: salaries, pensions, unemployment benefits and income from other economic activities. Income for the year 2007 is calculated in monthly terms, dividing annual income by 12 in the case of living people, and by the number of months of life in the case of the dead. One of the elements that can cause a spurious relationship between income and mortality is the reduction in income that in many cases occurs in the months prior to death as a result of illness. To avoid this effect, monthly income has also been obtained for the year 2006. The average of the two years has been calculated for each type of earnings and for total income. The income distribution (Figure 1) shows a right-skewed distribution. The individuals are grouped by deciles, adopting the standard that the first decile (0) corresponds to the richest individuals and the last (9) to the poorest individuals. 

### 2.4. Other Variables

The database provides a series of variables that have not been used in previous studies to analyze the relationship between the socio-economic status of workers and the mortality rate. These are extremely important for the analysis. 

Depending on the source of income, a set of dummy variables is constructed to classify the individuals depending on their economic and employment status. Individuals are considered as “LT-unemployed” if unemployment benefits represent 100% of their income (the long-term unemployed) and as “unemployed” if they received some unemployment benefits in 2006 or 2007.

The fact that a working-aged person receives a pension can be an indicator of health problems, which is considered important if the person has retired from the work force and the pension is the only source of income, “Tot-pension”. The dummy “Pension” indicates if the individual obtains additional income from some other sources (such as wages), meaning that the person is still working. Two additional dummies indicate whether or not there is any income obtained from economic activities, “Tot-rent” and “rent” (depending on being the economic rents the only source of income or not), as indicators of the capacity of individuals to self-manage their own work. 

For salary recipients, there is information on the type of employment contract. It distinguishes between temporary jobs lasting less than one year and permanent jobs. As an individual may have had different types of work over the year, the “labor” variable takes the value “0” if they only had a stable job, “1” represents precarious as well as stable jobs and “2” signifies precarious jobs only. 

The “disability” variable takes the value 0 for no disability or with a degree of disability of less than 33%; 1 if the disability is equal to or over 33% and less than 65%, and there is no reduction in mobility, and 2 in the rest of the cases. 

Table 3 presents some descriptive statistics that indicate how some of the variables of interest are related to income level. There are 43,070 people whose income comes exclusively from pensions; they are overrepresented in the lowest income brackets (deciles 7 to 9). Those who do not receive any type of pension are underrepresented in the poor deciles (6 to 9) and overrepresented in the others. People who combine pensions and other sources of income are grouped in deciles (6 to 8), the lower-middle income deciles. On the other hand, people who do not have any type of income from economic activities are underrepresented in the first deciles (especially in the first, the highest income decile). Those who share income from economic activities with other types of income are clearly located in the highest income strata. However, those whose only source of income is economic activities are polarized between the high-income and low-income groups.

The population with some type of disability accounted for 5.6% of the sample. It can be observed that while the absence of disability is distributed among all income groups (with a slight over-representation in deciles 0 and 1, and under-representation in deciles 7 and 8), the presence of a level of disability between 33% and 65% gains weight as income decreases, and the most severe level of disability shows a certain polarization with a high concentration especially in the poorest deciles (7 to 9) and a certain relative weight in deciles 1 and 2.

Two geographical variables are used: “Big-municipality” = 1 if the municipality of residence is larger than 40,000 inhabitants and “capital” = 1 if the residence is in the capital of the province. 

We have no information on the household size, but it can be approximated by the number of ascendants and descendants declared for tax purposes as living with the individual. “Descendants” identifies the number of descendants as 0, 1, 2, 3 and 4 (or more), and “Ascendants” = 1 if the person lives with any ascendant. The variable “Family-disability” is equal to 1 if any ascendant or descendant with whom the individual lives has any degree of disability. 

The “nationality” variable indicates Spanish nationals (0), those from any other European country (1), from Latin America (2), from Africa (3) and from the rest of the world (4).

### 2.5. The Statistical Model

Three logit models are estimated. The dependent variable takes the value 1 in the case of death and 0 otherwise, and the explicative variable is the decile of the individual’s income, in the form of a dummy variable for each one of the last 9 deciles. The first model (M1) includes the age, the sex and the relationship between income and sex. The coefficients are estimated and so are the probabilities of death by sex and decile, taking sex as a reference. The mortality ratios (MR) are compared with the average income levels for each decile, examining the linear or non-linear nature of the relationship. Subsequently, the second model (M2) is estimated, adding the variables of employment, social and geographical nature, thus obtaining a new mortality gradient on income and examining its variation with respect to M1. Lastly, a third model (M3) is built, including the level of education and its relationship to sex. The effects are analyzed, including the relationship between income-mortality and the inequality in the probability of death associated with the level of education. 

This estimation strategy allows us to analyze whether, as some of the literature on the subject suggests, the inclusion of other socioeconomic indicators attenuates the relationship between income and the probability of death. The strategy makes it possible to analyze changes in the influence of income on mortality by incorporating new variables, which is relevant for studying whether the effect of income and education is the same. Furthermore, it will be possible to calculate elasticities by observing whether the income-mortality relationship is linear or not.

## 3. Results 

### 3.1. The Estimated Coefficients

Table 4 presents the coefficients (odd-ratios) estimated for the three models. In model M1, all the parameters are statistically different from one, except some interactions between sex and income. The income gradient in the case of men is evident, and there are clear differences by sex in the relationship between income and the probability of death. 

The inclusion of socio-economic and geographical variables in the second model (M2) produces the following effects: (a) a reduction in the odds-ratio of the age for the 55–64 year old group, suggesting that other variables (such as pensions or disability) are capturing these effects; (b) the coefficients for all income dummies are lower, indicating an attenuation of the gradient, even though the gradient still remains; and (c) the interactions by sex increase slightly. 

The new set of variables offers statistically significant information. The probability of death is lower for non-Spaniards: by 35% in the case of European citizens and by almost 60% in the rest. Residence in urban areas does not show any differences, but residing in the capital of a province increases the probability of death by 14%. Only second-degree disability has a significant effect, multiplying the probability of death by 2.3. The precariousness of employment has consequences on mortality: if there is not any other type of employment, the probability of death increases by 33%, and if it coexists with a stable employment relationship, it increases by 14%. 

Obtaining income from other economic activities reduces the probability of death by 30%, independently of whether it is the only source of income or not. Receiving a pension, whether it is only a partial source of income or the only source of income, multiplies by two the probability of death. Unemployment has a dual effect, depending on whether it is temporary or of long duration. While persistent unemployment does not affect the probability of death, the episodic nature of unemployment reduces it by 22%. 

Including the level of education in the third model (M3) does not substantially alter the estimations of the coefficients associated with age, sex and the socio-economic variables of M2. This shows a strong stability but, once again, it contributes to reduce the size of the gradient very clearly, as far as men are concerned. The education gradient has a different profile depending on the sex of the individual. 

### 3.2. The Probabilities of Death and the Income Gradient

The income gradient in M1 (Table 5) is higher for men than for women. The mortality rate ratios (MRR) of men increase quickly in the first two deciles; this ratio levels off in the next two deciles and increases again after the median. The slope shows two differentiated sections: up to the median, reductions of 100 Euros of income increase the probability of death very little, and after that, it increases by more than 0.5 points per thousand. In women, the gradient only appears clearly for the 40% with less income, and reductions in mortality for each 100 Euros of income are around 0.18 points per million in groups 6–8. 

**Figure 2:**
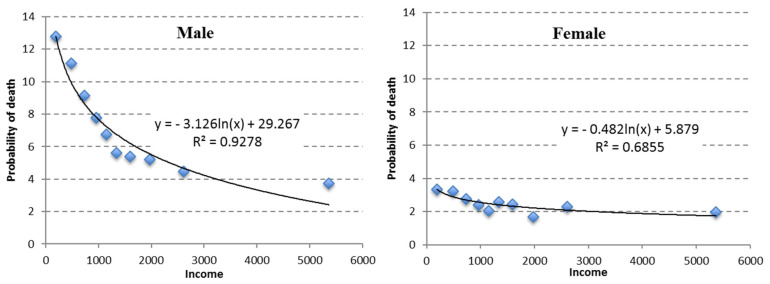
Estimated probability of death by income for males and females.

The point cloud in Figure 2 of the estimated probability of death by income suggests that in the case of a linear adjustment, it should have had two differentiated sections or slopes, one with a steep slope for low-income individuals and another with a more attenuated slope for those with higher incomes. The adjustment using a logarithm function is better than the linear function. The coefficient that accompanies the logarithm of income (−3.126 for men and −0.482 for women) is an indicator of the overall intensity of the relationship (divided by the corresponding level of income, it indicates the slope of the function). It is apparent that the gradient is non-linear and is more intense in the case of men than women. 

### 3.3. The Education Gradient

The MRR (Table 6) indicates that the level of education generates inequality in the case of men but not in the case of women. Completing obligatory secondary education reduces the probability of death in men by 4.8%. Nevertheless, reaching the level of post-secondary reduces it by 13.6% more, and for university-educated individuals by an additional 12.7%. 

### 3.4. The Income Gradient and the Expanded Models

**Figure 3:**
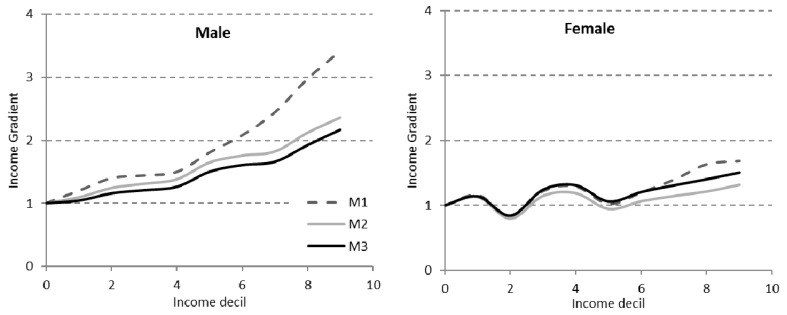
Income gradient in models 1, 2 and 3.

Controlling by socio-economic variables and level of education alters the income gradient (Table 7 and Figure 3). The inclusion of socio-economic variables reduces the mortality ratios for both men and women, with a greater intensity for those with less income, contributing to the reduction in the gradient. Taking into account the level of education has a different effect depending on the sex of the individual. In the case of men, the gradient attenuates in very similar percentages for all income levels. Nevertheless, in the case of women, including the level of education attenuates the importance of the income gradient and even more so the lower the income level.

## 4. Discussion

We have found inequalities in mortality depending on the income of the Spanish working-age population. The results are in line with those reached in similar studies in other countries in various ways, including (a) the very existence of the gradient (with a MRR between the group with less and more income of 3.4 in men and of 1.7 in women) [1,3,7,18]; (b) in its non-linear nature [13,20]; (c) in the attenuation of the gradient when considered together with other types of variables such as social, employment and geographical (reaching 36% in the case of men and 10.4% in that of women (M2 over M1) [1,5,6,11,14]. The attenuation of the gradient does not mean it disappears since the gradient remains with an RR of 2.2 for men and of 1.5 for women. Furthermore, controlling for the level of education attenuates the income gradient in men but in intensifies it for women. 

One of the objections often made to using income as a measure of SES is the inverse causality, especially among the working-age population. Poor health can lead to the total or partial abandonment of the job market and, as a consequence, a reduction in income. Reducing the inverse causality can be done by using family income instead of individual income and adjusting other socio-economic variables that precede the income. The CWLS does not allow working with family income data adjusted by size. However, it does allow other ways to control the inverse causality. First, using the average income of two years reduces the dependence on recent health episodes and provides more regular income flows. Secondly, controlling by socio-economic variables can serve to capture one part of the inverse causality.

Income from pensions is associated with early retirement or disability (indicator of poor health) and is usually lower than salaries (pensioners are overrepresented in deciles 6 to 9). The disability variable directly shows a health problem classified as such by medical services. Those of type 2 are especially concentrated in the lesser income deciles (7 to 9). Both variables, each separately, double the probability of death, which contributes to reducing the part of inequality attributable to income. Another strategy is to exclude those with disabilities given that including them skews upwards the coefficient of income. In our case (data not shown), the gradient is further reduced following the strategy of not excluding those individuals and including the variables that cover this situation. 

Another relevant variable is the way in which one enters the job market. The precariousness of employment increases the probability of death. It perhaps reflects the phenomenon of inverse causality because those with poor health find worse jobs, but it also reflects the fact that precariousness makes it necessary to accept jobs with greater risk of illnesses and accidents or that are costly in terms of quality of life. 

An interesting fact is that the probability of death is lower for non-Spaniards: by 35% in the case of European citizens and by almost 60% for the rest. Existing research on immigrants’ health in advanced countries has extensively reported a health advantage, which is frequently observed to diminish or even reverse with increasing time of residence [23,24,25,26]. This is the so called “Healthy Migrant Effect” that describes an empirically observed mortality advantage of migrants from certain countries of origin, relative to the majority population in the host countries. This empirical relationship has also been found to be true for the Spanish case [27].

Geographical variables allow us to conclude that there are no substantial differences between living in a city of more than 40,000 inhabitants than in another smaller city but, nevertheless, living in a capital city increases the probability of death by 14%. The inclusion of this variable can contribute to strengthening the role of income in the explanation of inequality since the inhabitants of the capital city are overrepresented, especially in the high-income deciles. 

Using a model with levels of education interacting with the sex of the individual as the only variable and controlled by age, the MRRs are closer to other previous studies that also suggest that the inequality in the level of education is less for women than for men, and is even non-existent in groups of more advanced age [5,9]. These differences between women and men can be attributed to the fact that some studies analyze the whole population, while we restrict it to the population included in the CWLS (of special importance to women). The simultaneous use of income and level of education reduces both gradients in the case of men, splitting the inequality effect between both dimensions. In the case of women, it almost cancels out the education gradient in its entirety, but accentuates the role of income as a source of inequality.

## 5. Conclusions

There seems to be a strong consensus on the existence of a relationship between socioeconomic status (SES) and mortality. The analysis of the impact of socioeconomic inequalities on mortality is relatively new in Spain, and existing studies are limited with regard to the dimensions of the SES indicators used and the geographical area covered: (1) the studies are limited to urban areas or refer to specific regions and (2) they utilize the level of education as the only socioeconomic indicator. The main reason for this limitation is the data sets previously used: mainly Census records and mortality data from the National Institute of Statistics. We overcome this problem by using the Continuous Working Life Sample, which includes a wide range of socio-economic and geographical information. However, it should be noted that the data used also have some limitations. First, they do not include those workers who in the reference year are unemployed and do not receive any type of income or subsidy. However, the main limitation comes from the fact that contributors to other welfare systems, such as civil servants, military personnel and justice administration personnel, do not appear either. This may lead to an underrepresentation in the sample of the population with a higher level of education. Nevertheless, the analysis of the sample offers some interesting results that shed some light on the relationship between mortality and income.

We have estimated the probabilities of death for each income decile, and the mortality rate ratios in three different models: (1) using only income, controlled by age and sex, (2) adding socio-economic and geographical variables, and (3) adding level of education. As a result, the association of the relationship between income and mortality is verified, as well as its non-linear nature, the greater intensity for men than for women, and its persistence when controlling for variables that try to capture causality. A gradient appears in the mortality rate over income deciles with a maximum MRR of 3.41 in men and 1.68 in women. The shape of the adjustment is clearly non-linear and levels out as income increases. The gradient attenuates for both sexes when new SES variables are introduced. 

The main results of the analysis are as follows: (a) the very existence of the gradient (with a MRR between the group with less and more income of 3.4 in men and of 1.7 in women); (b) its non-linear nature; (c) the attenuation of the gradient when considered together with other types of variables such as social, employment and geographical; (d) the attenuation does not mean it disappears since the gradient remains; and (e) controlling for the level of education attenuates the income gradient in men but it intensifies it for women. 

In summary, the association of the relationship between income and mortality in Spain is verified, as well as its non-linear nature. Much of the literature has focused on whether the relationship between mortality and income is linear or not. The question is not trivial, since a linear relationship would imply that an increase in income in low-income groups generates a reduction in their mortality rate similar to the increase in mortality that a parallel reduction in income would cause in high-income sectors, so that income redistribution policies would have no effect on overall mortality. In contrast, the non-linear relationship, as is the case here, suggests that income redistribution resulting from progressive taxation systems would lead to higher reductions in mortality for low-income groups than the reductions induced in the mortality of the high-income population, thus reducing overall mortality.

## Figures and Tables

**Table 1:** Sample distribution and mortality rate by age and sex.

	Sample Characteristics ^a^			Mortality Rate ^b^		
	Male	Female	Total	Male	Female	Total
25–34	17.4 (18.6)	15.1 (15.2)	32.5 (33.8)	1.14	0.50	0.84
35–44	16.7 (17.9)	13.0 (13.5)	29.7 (31.4)	2.74	1.37	2.14
45–54	12.9 (13.7)	9.30 (9.7)	22.2 (23.3)	8.42	3.21	6.23
55–64	9.75 (7.4)	5.9 (4.1)	15.7 (11.5)	21.70	8.72	16.81
Total	56.7 (57.6)	43.3 (42.4)	100 (100)	6.80	2.46	4.92

^a^ Percentage over total sample. Within brackets: Spanish Labor Force Survey (EPA 2007); ^b^ Mortality rate per 1000 people.

**Table 2:** Educational level by age group.

Age	University Degree	Post-Secondary	Secondary Education	Primary and Illiterates	Total
25–35	6.58	33.81	40.15	19.47	100
35–45	8.8	35.5	37.99	17.71	100
45–55	6.31	28.62	34.96	30.1	100
55–65	4.49	17.97	28.5	49.04	100
Total	5.72	25.46	31.69	37.13	100

**Table 3:** Percent distribution of people with disabilities, and various forms of income across income deciles.

	0	1	2	3	4	5	6	7	8	9	Total
Disability											
No disability	10.2	10.1	10.1	10.1	10.1	10.1	10.0	9.8	9.6	10.0	100
More than 33%	4.3	6.2	8.3	8.2	7.2	7.6	10.2	16.7	22.2	9.1	100
More than 65%	8.5	11.5	12.5	10.9	8.0	8.7	8.1	11.5	9.9	10.4	100
Income from Pensions											
No-pension	10.5	10.4	10.3	10.4	10.5	10.4	10.0	9.3	8.7	9.5	100
Pension and other	9.8	10.2	10.2	9.2	8.9	9.2	11.3	13.4	11.5	6.3	100
Tot-pension	1.4	2.7	3.1	3.7	3.4	4.0	6.9	14.5	31.7	28.6	100
Income from economic activities											
No rent	7.7	9.5	10.0	10.3	10.5	10.6	10.6	10.4	10.5	10.0	100
Rent and other	25.0	14.8	11.2	8.9	7.3	6.1	6.5	7.7	6.9	5.7	100
Tot-rent	32.8	10.3	7.0	5.0	3.5	3.4	3.8	5.3	5.8	23.0	100

**Table 4:** Estimated odd-ratios for Models 1, 2 and 3.

	M1		M2		M3	
VARIABLES	Coeff	CI	Coeff	CI	Coeff	CI
Age = 25–34 (ref. categ.)	1.00		1.00		1.00	
Age = 35–44	2.69 ***	2.26–3.18	2.75 ***	2.31–3.26	2.76 ***	2.32–3.27
Age = 45–54	7.88 ***	6.73–9.22	7.56 ***	6.44–8.86	7.51 ***	6.40–8.81
Age = +55	19.8 ***	17.0–23.0	13.9 ***	11.8–16.1	13.5 ***	11.5–15.8
Sex = woman	0.53 ***	0.37–0.74	0.47 ***	0.33–0.66	0.54 **	0.33–0.86
income-decile = 0-high (ref. categ.)	1.00		1.00		1.00	
income-decile = 1	1.20 **	1.00–1.42	1.09	0.91–1.30	1.04	0.87–1.24
income-decile = 2	1.40 ***	1.18–1.66	1.25 **	1.04–1.48	1.16	0.97–1.38
income-decile = 3	1.45 ***	1.21–1.72	1.31 ***	1.10–1.57	1.21 **	1.00–1.45
income-decile = 4	1.50 ***	1.25–1.79	1.39 ***	1.16–1.66	1.27 **	1.05–1.52
income-decile = 5	1.82 ***	1.52–2.16	1.66 ***	1.39–1.98	1.51 ***	1.25–1.81
income-decile = 6	2.10 ***	1.75–2.50	1.78 ***	1.47–2.13	1.62 ***	1.34–1.95
income-decile = 7	2.47 ***	2.08–2.93	1.84 ***	1.53–2.21	1.67 ***	1.39–2.01
income-decile = 8	3.02 ***	2.57–3.54	2.15 ***	1.80–2.57	1.95 ***	1.62–2.33
income-decile = 9-low	3.49 ***	2.96–4.09	2.41 ***	2.01–2.87	2.20 ***	1.83–2.63
woman +1.income-decile	0.97	0.61–1.52	1.03	0.65–1.63	1.10	0.69–1.73
woman +2.income-decile	0.60 **	0.36–0.98	0.64 *	0.39–1.05	0.72	0.43–1.19
woman +3.income-decile	0.85	0.53–1.33	0.87	0.55–1.38	1.03	0.64–1.63
woman +4.income-decile	0.86	0.54–1.37	0.86	0.53–1.36	1.04	0.64–1.67
woman +5.income-decile	0.56 **	0.35–0.91	0.57 **	0.35–0.91	0.70	0.42–1.14
woman +6.income-decile	0.58 **	0.36–0.89	0.60 **	0.38–0.93	0.75	0.47–1.18
woman +7.income-decile	0.57 ***	0.37–0.86	0.62 **	0.40–0.94	0.78	0.50–1.21
woman +8.income-decile	0.54 ***	0.36–0.80	0.56 ***	0.38–0.83	0.72	0.47–1.09
woman +9.income-decile	0.48 ***	0.32–0.72	0.55 ***	0.36–0.81	0.67 *	0.45–1.04
nationality = Spanish (ref. categ.)			1.00		1.00	
nationality = European			0.65 ***	0.47–0.88	0.66 ***	0.48–0.88
nationality = Latin-American			0.42***	0.28–0.61	0.42 ***	0.28–0.62
nationality = African			0.41 ***	0.25–0.66	0.40 ***	0.24–0.64
nationality = Other			0.34 **	0.12–0.90	0.33 **	0.12–0.89
Big-municipality			0.97	0.88–1.06	0.97	0.88–1.06
Capital			1.14 ***	1.03–1.26	1.16 ***	1.05–1.28
Disabil: no disability (ref. categ.)			1.00		1.00	
Disabil: 33–65%			0.96	0.85–1.07	0.95	0.84–1.06
Disabil: +65%			2.34 ***	1.86–2.95	2.36 ***	1.87–2.96
Labor-stable job (ref.cat.)			1.00		1.00	
Labor-mix-contracts			1.14 **	1.02–1.27	1.14 **	1.02–1.27
Labor- precarious			1.33 ***	1.16–1.52	1.33 ***	1.16–1.51
Rents: no-rents (ref.cat.)			1.00		1.00	
Rents: some rents			0.68 ***	0.59–0.78	0.69 ***	0.60–0.78
Rents: Tot-rent			0.67 ***	0.53–0.83	0.66 ***	0.53–0.83
Pensions: no-pension (ref. categ.)			1.00		1.00	
Pensionns: some pension			2.09 ***	1.87–2.31	2.07 ***	1.86–2.30
Pensions: Tot-pension			1.96 ***	1.68–2.27	1.96 ***	1.68–2.27
Unemployed			0.78 ***	0.70–0.87	0.78 ***	0.70–0.87
Long Term-unemployed			1.01	0.82–1.24	1.02	0.83–1.25
descendants = 0 (ref. categ.)			1.00		1.00	
descendants = 1			0.87 **	0.78–0.97	0.87 **	0.78–0.97
descendants = 2			0.73 ***	0.63–0.83	0.73 ***	0.63–0.83
descendants = 3			0.67 **	0.49–0.91	0.67 **	0.49–0.91
descendants = +3			0.91	0.51–1.61	0.91	0.51–1.61
Ascendants			0.70	0.42–1.14	0.70	0.42–1.14
Fam-disability			0.83	0.54–1.28	0.83	0.54–1.27
Edu: University (ref. categ.)					1.00	
Edu: Post-secondary					1.15	0.90–1.45
Edu: Secondary					1.33 **	1.05–1.68
Edu: Primary					1.40 ***	1.11–1.77
woman+ post-secondary educ.					1.01	0.66–1.55
woman+ secondary educ.					0.61 **	0.40–0.94
woman+ primary or less educ.					0.66 *	0.43–1.01
Constant	0.0006 ***	0.0005–0.0008	0.0007 ***	0.0006–0.0010	0.0006 ***	0.0005–0.0008

*** *p* < 0.01, ** *p* < 0.05, * *p* < 0.1.

**Table 5:** Income, probability of death and MRR for M1.

		MALE				FEMALE			
Decil	Income ^a^	Probability of Death ^b^		MRR	Δ*p*/Δy ^c^	Probability of Death		MRR	Δ*p*/Δy
0	5362.9	3.74	3.29–4.18	1.00		1.99	1.35–2.62	1.00	
1	2611.1	4.48	3.92–5.03	1.20	−0.027	2.31	1.69–2.93	1.16	−0.012
2	1975.5	5.22	4.58–5.86	1.40	−0.117	1.67	1.12–2.22	0.84	0.101
3	1598.4	5.38	4.70–6.07	1.44	−0.043	2.43	1.78–3.09	1.23	−0.203
4	1344.7	5.59	4.87–6.31	1.50	−0.083	2.57	1.84–3.29	1.29	−0.053
5	1151.5	6.75	5.90–7.60	1.81	−0.598	2.04	1.42–2.66	1.03	0.273
6	959.9	7.77	6.78–8.76	2.08	−0.533	2.39	1.80–2.98	1.20	−0.183
7	732.5	9.13	8.05–10.21	2.44	−0.600	2.78	2.21–3.34	1.40	−0.170
8	485.5	11.12	9.97–12.27	2.98	−0.804	3.25	2.77–3.73	1.64	−0.191
9	195.7	12.77	11.43–14.12	3.42	−0.570	3.35	2.80–3.89	1.69	−0.034

^a^ Average monthly income; ^b^ Probability of death (×1000) and 95% interval of confidence; ^c^ Slope (Δ*p*/Δy): variation in the probability of death for every 100€ of change in income.

**Table 6:** Probability of death and MRR by sex and education.

	Male			Female		
Education	Probability ^a^	CI	MRR	Probability ^a^	CI	MRR
0	5.59	4.36–6.81	1	2.48	1.67–3.29	1
1	6.40	5.80–7.00	1.15	2.89	2.47–3.31	1.16
2	7.41	6.92–7.90	1.33	2.04	1.75–2.32	0.82
3	7.78	7.33–8.24	1.39	2.31	2.01–2.61	0.93

^a^ Probability of death (×1000).

**Table 7:** Income gradient for models 1, 2 and 3 (M1, M2, M3).

	Mortality Rate Ratios for Males (MRR)					Mortality Rate Ratios for Females (MRR)				
Decil	M1	M2	M3	M2/M1	M3/M2	M1	M2	M3	M2/M1	M3/M2
0	1.000	1.000	1.000			1.000	1.000	1.000		
1	1.198	1.092	1.040	−8.8	−4.8	1.163	1.131	1.139	−2.7	0.7
2	1.397	1.243	1.156	−11.1	−7.0	0.840	0.799	0.839	−4.9	4.9
3	1.440	1.310	1.206	−9.0	−8.0	1.225	1.149	1.243	−6.2	8.2
4	1.497	1.382	1.261	−7.6	−8.8	1.293	1.190	1.312	−8.0	10.3
5	1.806	1.649	1.499	−8.7	−9.1	1.028	0.947	1.058	−7.8	11.7
6	2.079	1.759	1.606	−15.4	−8.7	1.204	1.066	1.207	−11.4	13.2
7	2.444	1.825	1.659	−25.3	−9.1	1.399	1.148	1.310	−17.9	14.2
8	2.976	2.124	1.924	−28.6	−9.4	1.636	1.216	1.406	−25.7	15.6
9	3.417	2.366	2.166	−30.8	−8.4	1.685	1.319	1.509	−21.7	14.4

M2/M1: Percentage of MRR reduction of M2 over M1; M3/M2: Percentage of MRR reduction of M3 over M2.

## Data Availability

Publicly available datasets were analyzed in this study. This data can be found here: https://www.seg-social.es/wps/portal/wss/internet/EstadisticasPresupuestosEstudios/Estadisticas/EST211 (accessed on 31 August 2021).
